# Highly Stabilized Core-Satellite Gold Nanoassemblies in Vivo: DNA-Directed Self-Assembly, PEG Modification and Cell Imaging

**DOI:** 10.1038/s41598-017-08903-0

**Published:** 2017-08-17

**Authors:** Liangfeng Tang, Guiping Yu, Lishan Tan, Min Li, Xiulong Deng, Jianyu Liu, Aiqing Li, Xuandi Lai, Jianqiang Hu

**Affiliations:** 1 Key Laboratory of Fuel Cell Technology of Guangdong Province, Department of Chemistry, College of Chemistry and Chemical Engineering, South China University of Technology, Guangzhou, 510640 China; 2 State Key Laboratory of Organ Failure Research, National Clinical Research Center for Kidney Disease, Nanfang Hospital, Southern Medical University, Guangzhou, 510515 China; 3 State Key Laboratory of Pulp and Paper Engineering, South China University of Technology, Guangzhou, 510640 China

## Abstract

Au nanoparticles (NPs) have important applications in bioimaging, clinical diagnosis and even therapy due to its water-solubility, easy modification and drug-loaded capability, however, easy aggregation of Au NPs in normal saline and serum greatly limits its applications. In this work, highly stabilized core-satellite Au nanoassemblies (CSAuNAs) were constructed by a hierarchical DNA-directed self-assembly strategy, in which satellite Au NPs number could be effectively tuned through varying the ratios of core-AuNPs-ssDNA and satellite-AuNPs-ssDNAc. It was especially interesting that PEG-functionalized CSAuNAs (PEG-CSAuNAs) could not only bear saline solution but also resist the enzymatic degradation in fetal calf serum. Moreover, cell targeting and imaging indicated that the PEG-CSAuNAs had promising biotargeting and bioimaging capability. Finally, fluorescence imaging *in vivo* revealed that PEG-CSAuNAs modified with N-acetylation chitosan (CSNA) could be selectively accumulate in the kidneys with satisfactory renal retention capability. Therefore, the highly stabilized PEG-CSAuNAs open a new avenue for its applications *in vivo*.

## Introduction

Gold nanoparticles (NPs) have been attracting increased attention because they have easy surface modification and unique physical and chemical properties^1, 2^ and biomedical applications in luminescence imaging^3^, drug carrier^4^, tumor targeting^5, 6^, cancer therapeutics^7^ and radiotherapy enhancer^8^. Nevertheless, single Au NPs have limited drug loading efficiency, easy aggregation in serum and strong electrolyte solution and potential biotoxicity, which greatly limits their medical application. The potential toxicity *in vivo* closely associates with its characteristics such as size, shape and surface properties^9–12^. For example, Niidome *et al*. found that cetyltrimethylammonium bromide (CTAB)-coated gold nanorods were highly cytotoxic at as low as 0.05 mM (80% of dead cells) and when CTAB was replaced by thiol-functionalized poly(ethylene glycol) (PEG-SH), 95% of viable cells were observed even though the nanorod concentration was up to 0.5 mM^13^. Compared with isolated Au NPs, Au nanoassemblies (NAs) offer tailored and enhanced optical and electrical properties and have great potential in molecular recognition, plasmon-enhanced fluorescence, nanobiotechnology and nanomedicine^14^. Capasso’s group has successfully assembled heteropentamer clusters through using DNA-functionalized nanoparticles, which can be fabricated into plasmon-enhanced biomolecular detector^15^. Moreover, it is also promising to improve drug loading amount, luminescence imaging efficiency and biological compatibility and stability in comparison with Au NPs. For example, Koyakutty’ group reported that folic-acid-conjugated fluorescent Au_25_ nanoclusters showed excellent stability over a wide range of pH from 4 to 14 and fluorescence efficiency of ~5.7% at pH 7.4 in phosphate buffer saline (PBS) and effective protection of nanoclusters by serum albumin and outlooked that the bio-friendly nature, near-infrared fluorescence and receptor specific cancer targeting ability of Au clusters would make them an ideal candidate for optical-imaging-based cancer detection^16^. What’ more, Sharma *et al*. demonstrated recently that equilibrium encapsulation of 1 nm hydrophobically modified AuNPs occurs at the rim and resulted in formation of a ring of gold and showed that lipid nanodiscs can enhance gold cluster formation, which can be further exploited in imaging applications^17^.

DNA has been regarded as one of the most powerful biomolecules to control over the assembly architectures owning to its unique programmability, outstanding biocompatibility, ease of synthesis and modification as well as strong physicochemical stability^18–21^. At present, different structures of DNA-directed Au NAs have been reported, such as one-dimensional (1D) NP chains, 2D nanofilms and 3D star- or flower-like structures^13, 22–26^. But, DNA-directed Au NAs are still rare to be applied in biomedine *in vivo* although Au NAs have many advantages such as more drug loading amount, higher luminescence imaging efficiency and better biological compatibility and stability in comparison with isolated Au NPs. This is mainly because the stability of the existing Au NAs is still not enough to endure destruction from *in vivo* saline solution and serum containing abundant charged proteins, enzymes and nuclease^27–30^. To broaden the biological application of DNA-directed Au NAs, it is of key importance to fabricate Au NAs of nontoxicity and high stability in saline solution and serum^31^. It have been found that hydrophilic PEG moieties on the particle surface created steric hindrance for serum protein adsorption and slow down NP uptake by spleen^32^. And recently, Knop’ group has demonstrated that suitable length PEG can reduce non-specific interactions with biomeolecules and cells^33^.

In this study, we designed a well-defined core-satellite-shaped Au NAs (CSAuNAs) based on the hybridization of two complementary ssDNA, the stability including salt-bearable and serum-resisting ability, fluorescence imaging as well as targeted therapy application of which were investigated. The CSAuNAs were prepared through using core AuNPs of about 18.7 nm and satellite AuNPs of about 3.9 nm conjugated with two complementary ssDNA (i.e., ssDNA and ssDNAc), respectively. It was found that the molar ratio of core-AuNPs-ssDNA to satellite-AuNPs-ssDNAc played important roles in adjusting “satellite” quantities of the CSAuNAs. The CSAuNAs prepared at the molar ratio of 1:160 between core-AuNPs and satellite-AuNPs had very high stability and could bear 3-fold concentration of normal saline. Cytotoxicity and fluorescence-labeling characteristic of PEG-modified CSAuNAs were also explored. Cell experiments revealed that the PEG-modified CSAuNAs could maintain ≥90% cell viability within 5~50 µM. Fluorescence measurement indicated that the fluorescein isothiocyanate (FITC)-labeled PEG- and chitosan-modified CSAuNAs could effectively target toward renal tubular epithelial cells. Furthermore, fluorescence imaging of vital organs of mice revealed that PEG-CSAuNAs modified on NACS could be relatively more distribute in the kidneys than other organs after intravenous injection. Thus, NACS-modified PEG-CSAuNAs may provide an excellent opportunity for *in vivo* imaging and targeted therapy toward chronic kidney diseases.

## Results and Discussion

### Self-assembly of the CSAuNAs

**Figure 1:**
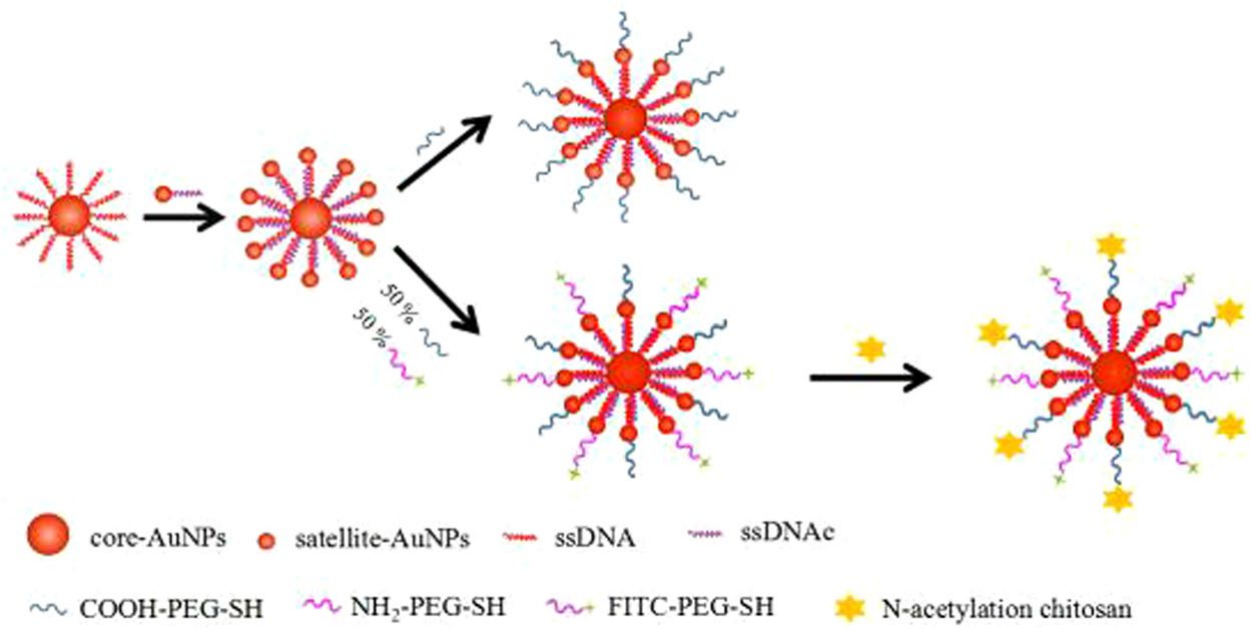
Schematic illustration of the DNA-directed self-assembly and surface modification of the “core-satellite”-shaped Au nanoassemblies.

In our strategy, highly stabilized CSAuNAs were constructed by a hierarchical DNA-directed self-assembly strategy, in which core AuNPs were encircled by satellite AuNPs. The schematic illustration of the DNA-directed self-assembly and surface modification of the CSAuNAs is presented in Fig. 1. First, Au NPs with the average diameters of around 18.7 nm and 3.9 nm were produced, respectively (Fig. S1). Subsequently, the Au NPs were reacted with complementary DNA (Table S1) and separated to form AuNP/DNA conjugates which would be used as CSAuNAs building blocks. To ensure the self-assembly, core AuNPs were conjugated with ssDNA at a molar ratio of 1:200 and satellite AuNPs were coupled with ssDNAc at 1:1. And then different amounts of satellite NPs self-assembled to core NPs through base-pairing between ssDNA and ssDNAc. Next, polyethylene glycol with thiol and carboxy group (HS-PEG-COOH) was modified to further improve the biological stability of CSAuNAs and reduce their non-specific interactions with biomolecules and cells. Meanwhile, the FITC-labeled PEG-modified CSAuNAs were also prepared by adding HS-PEG-COOH and HS-PEG-FITC (1:1 molar ratio). Finally, the soluble NACS was modified onto the HS-PEG-COOH of the FITC-labled PEG-modified CSAuNAs to target renal tubular epithelial cells.

### Transmission electron microscopy (TEM) images and gel electrophoresis of the CSAuNAs

**Figure 2:**
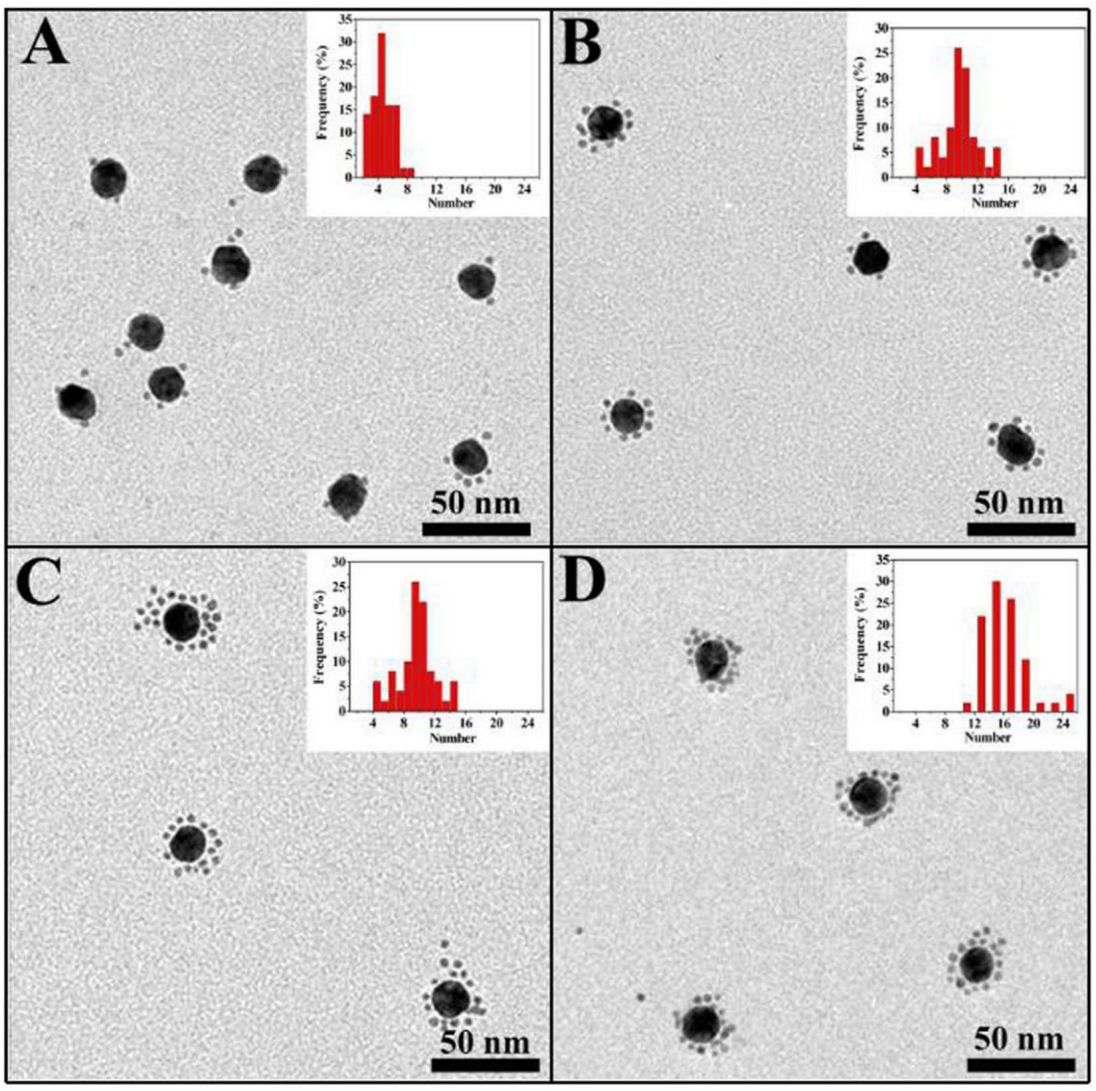
TEM images and corresponding histogram plots of the CSAuNAs assembled by the ratios between core-AuNPs-ssDNA and satellite-AuNPs-ssDNAc of (**A**) 1:40, (**B**) 1:80, (**C**) 1:160 and (**D**) 1:320.

Figure 2 shows TEM images and corresponding histogram plots of the CSAuNAs assembled by the different ratios between core-AuNPs-ssDNA and satellite-AuNPs-ssDNAc. It could be clearly seen that the “core-satellite” structure was successfully obtained by the present assembly strategy and the number of satellite NPs was adjustable through varying the molar ratio between satellite-AuNPs-ssDNAc and core-AuNPs-ssDNA. With increasing the molar ratio, the numbers of the satellite NPs presented regular variation, indicating that the molar ratio between satellite-AuNPs-ssDNAc and core-AuNPs-ssDNA played an important role in the self-assembly of the CSAuNAs. When the molar ratio increased from 40 to 160, the numbers of satellite NPs gradually increased from *ca*. 4 to *ca*. 16. Yet, when the molar ratio was more than 160 (*e.g*., 320), the number of the satellite NPs remained unchanged, which was probably because the number of the satellite NPs on the surface of the core NPs reached saturation. This was also demonstrated by gel electrophoresis measurement (Fig. S2). The CSAuNAs had slower migration rates than those of satellite Au NPs and core Au NPs, indicating the formation of the the “core-satellite” structure. Moreover, the more satellite NPs number had the slower migration rates.

### Surface modification and fluorescence labeling of the CSAuNAs and AuNPs

**Figure 3:**
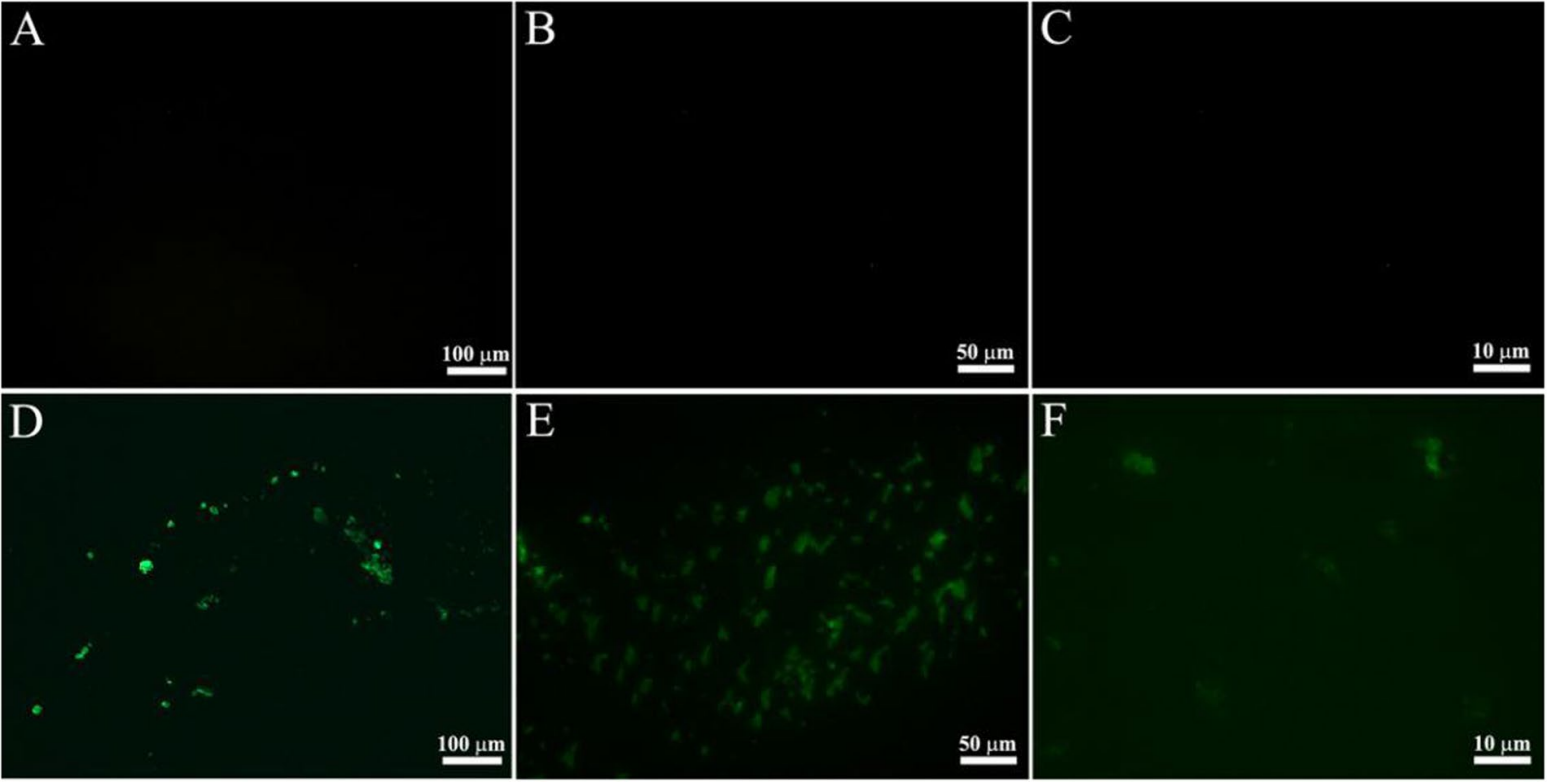
Fluorescence images of the PEG-CSAuNAs (**A**–**C**) before and (**D**–**F**) after the labeling of FITC. The images were obtained at different magnifications of (**A**,**D**) 200×, (**B**,**E**) 400× and (**C**,**F**) 1000×. Excitation light was 488 nm.

Many studies have demonstrated that PEG covering could minimize the toxicity of nanomaterials and improve its ability to resist immunological recognition, and then prolonged its vivo circulation time^34, 35^. The CSAuNAs were conjugated with HS-PEG-COOH through Au-S covalent bond. After conjugation, the hydrodynamic size of the CSAuNAs increased from 47.0 to 66.0 nm. Meanwhile, after the PEG modification, the zeta potential of the CSAuNAs changed from around -27 to around -1.2 mV (Fig. S3). The increase of hydrodynamic size and decrease of negative charge of the CSAuNAs revealed that PEG was successfully modified onto the surface of the CSAuNAs^36^. To perform cell fluorescence imaging, HS-PEG-FITC that was conjugated through using HS-PEG-NH_2_ and FITC was modified on the surface of the CSAuNAs. Figure 3 gives fluorescence images of the CSAuNAs conjugated with/without FITC. A clear yellow-green fluorescence clearly discerned in Fig. 3D–F indicated that FITC was successfully modified on the surface of the PEG-CSAuNAs. Similarly, AuNPs were able to be modified with PEG and FITC according to the same process above.

### Salt-bearable property of the PEG-CSAuNAs

**Figure 4:**
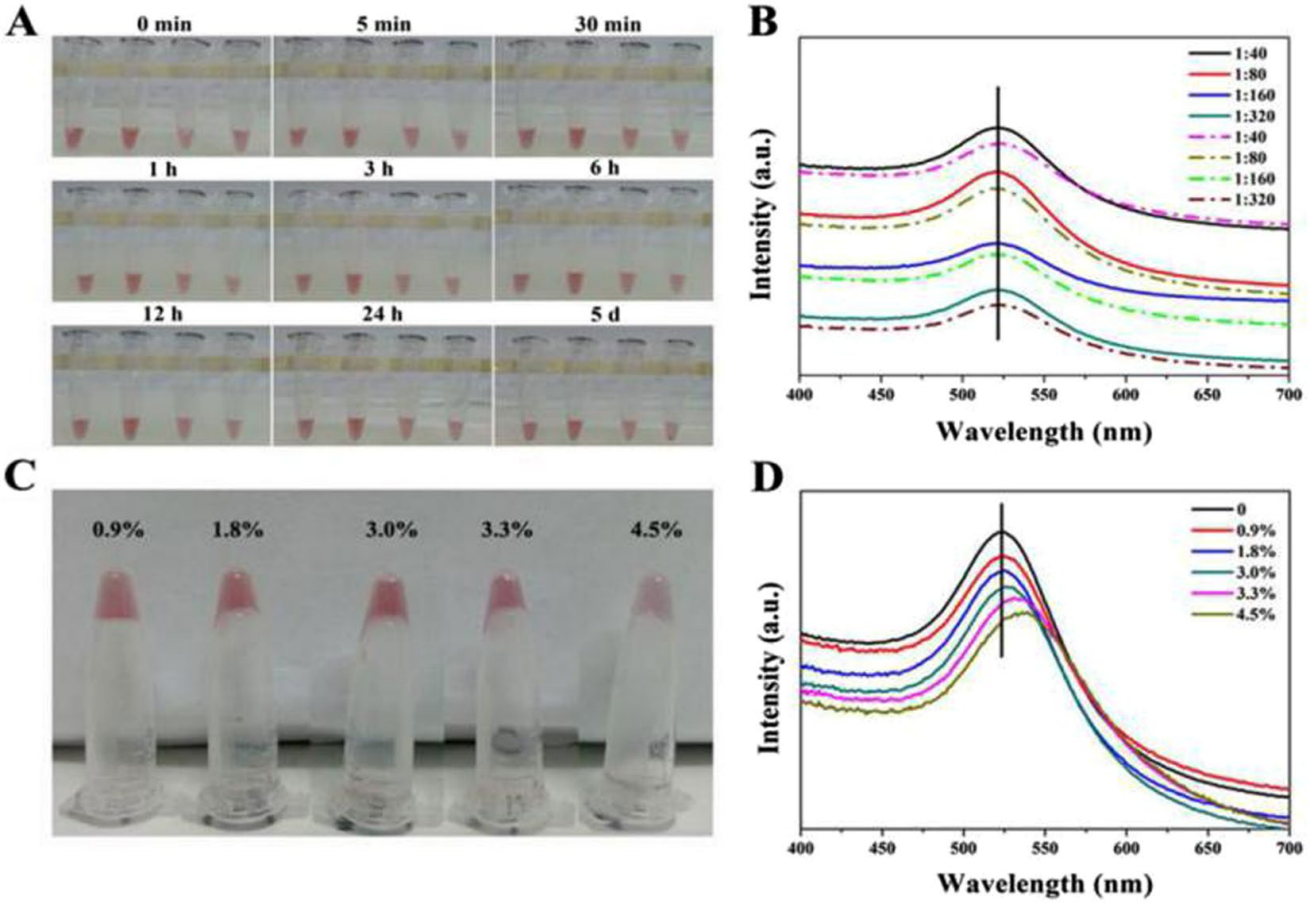
Stability evaluation of the PEG-CSAuNAs prepared at different molar ratios of core-AuNPs-ssDNA to satellite-AuNPs-ssDNAc in different NaCl solutions. (**A**) Digital photos of the PEG-CSAuNAs prepared at the 1:40, 1:80, 1:160 and 1:320 molar ratios (from left to right) after incubation with normal saline for different time. (**B**) UV-vis spectra of PEG-CSAuNAs prepared at the 1:40, 1:80, 1:160 and 1:320 molar ratios before (solid line) and after (dash line) incubation with normal saline for 5 days. (**C**) Digital photos and (**D**) UV-vis spectra of the PEG-CSAuNAs prepared at the 1:160 molar ratio in different NaCl concentrations for 2 h.

It is well known that the internal environment *in vivo* is essentially a salt solution equal to 0.9% normal saline, in which NPs easily aggregate due to electrostatic interaction^37^. To investigate the salt-bearable property of the PEG-CSAuNAs, different PEG-CSAuNAs were mixed with different NaCl concentrations, the aggregation states of which were observed through color change and peak shift of UV-vis absorption. Figure 4A depicts the color change of the PEG-CSAuNAs after incubation with normal saline for different time. It could be seen that the four PEG-CSAuNAs solutions still presented red color after 12 h, which was completely different from the light violet color of PEG-modified core AuNPs incubated with normal saline (Fig. S4). This suggested that the PEG-CSAuNAs prepared by the present assembly strategy had good stability in normal saline within 12 h. Nevertheless, it was important to note that there existed tiny sediment when the PEG-CSAuNAs prepared using the 1:320 molar ratio was stored in normal saline for 24 h although its supernatant still remained red color.

It is a sensitive tool for UV-vis spectroscopy to monitor whether Au NPs aggregate in different medium because the UV-vis absorption peak will red-shift with Au NPs aggregation^38^. As Fig. 4A,B display, the maximum absorption intensities for the PEG-CSAuNAs supernatants decreased to some degree after 5-day incubation with normal saline, indicating partial aggregation of the PEG-CSAuNAs. Nevertheless, there was also no obvious red-shift of the maximum absorption peak compared with PEG-CSAuNAs before incubation. Moreover, the PEG-CSAuNAs prepared using the 1:160 molar ratio should have the best stability in normal saline among these PEG-CSAuNAs (Fig. S5). To further study its salt-bearing ability, the PEG-CSAuNAs prepared using the 1:160 molar ratio were incubated with NaCl solution with different PEG-CSAuNAs (Fig. S5). To further study its salt-bearing ability, the PEG-CSAuNAs prepared using the 1:160 molar ratio were incubated with NaCl solution with different concentrations for 2 h (Fig. 4C,D). When the NaCl concentrations increased from 0.9% to 3.0%, the PEG-CSAuNAs still presented red color and the absorption peaks in UV-vis spectra showed no obvious red-shift. However, if the NaCl concentration increased up to 3.3%, the PEG-CSAuNAs would transform into purple and its UV-vis absorption peak presented approximately 7-nm-red-shift (Fig. S6). This revealed that the PEG-CSAuNAs could bear salt concentration as high as 3.0% (more than 3-fold of that in normal saline), thus further demonstrating that the salt-bearing stability of the Au NPs were largely enhanced because of its self-assembly structure and surface optimization.

### Serum-resisting properties of the PEG-CSAuNAs

**Figure 5:**
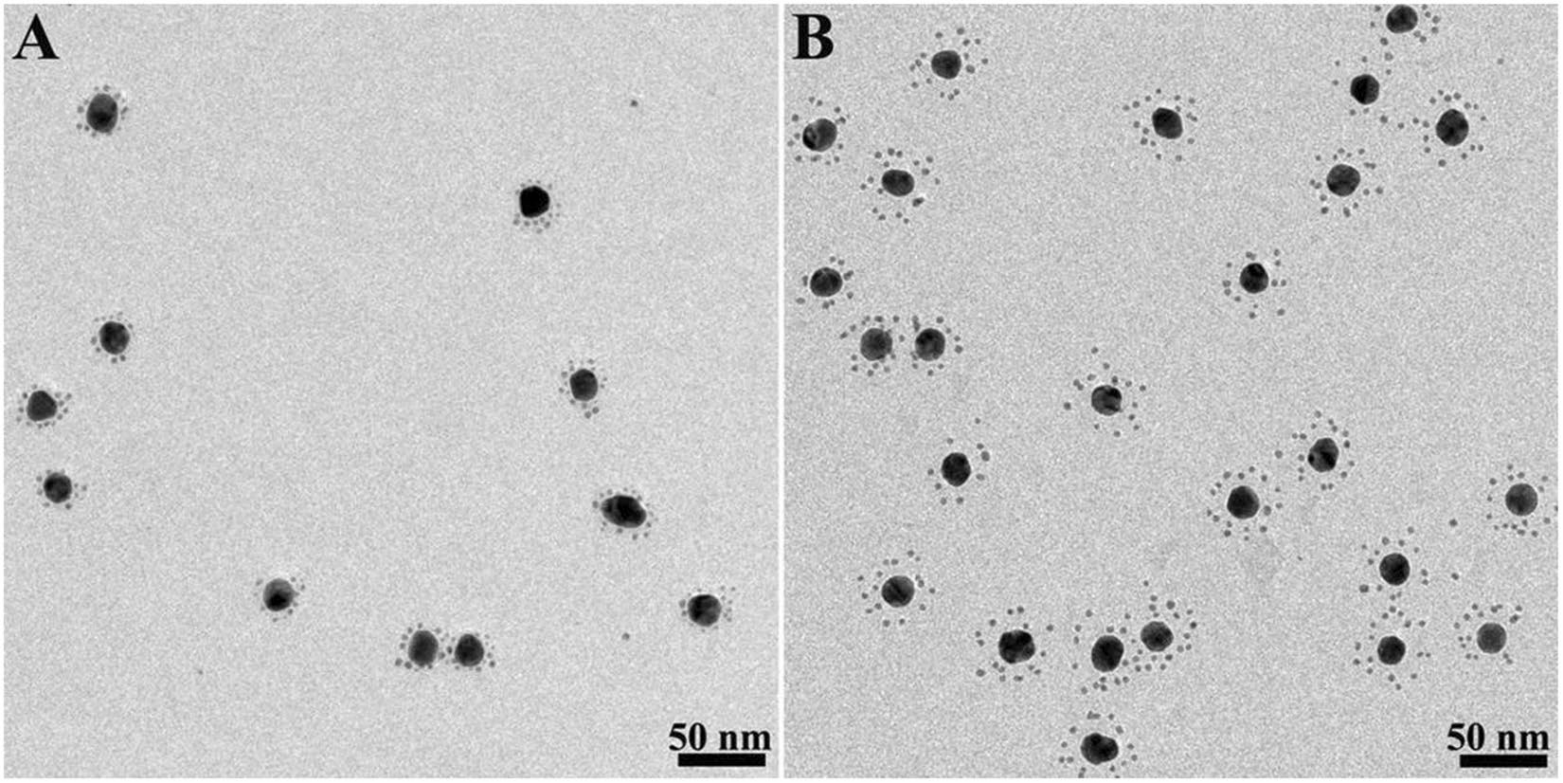
TEM images of the PEG-CSAuNAs prepared at the 1:160 molar ratio (**A**) before and (**B**) after incubation with 10% FCS for 6 h.

It is known that serum contains a variety of enzyme including nuclease which will cause the degradation of exogenous oligonucleotide^39^. The PEG-CSAuNAs prepared at the 1:160 molar ratio were incubated with 10% fetal calf serum (FCS) for 6 h and characterized by TEM (Fig. 5). The yield of PEG-modified Au NAs with satellite structures was almost 95% before incubation and nearly 90% after incubation. Enumerating from TEM imagines with more core-satellite structures (Fig. S7), the average satellite quantity of the PEG-CSAuNAs was counted to be about 15, which was almost the same as that before incubation. We also found that the average diameter of core nanoparticles was 18.7 ± 1.3 nm before incubation and were almost 18.7 nm after incubation. Besides, the “core-satellite” structure was well maintained although the “core-satellite” structure of the PEG-CSAuNAs incubated with FCS presented an obvious increase from 4.5 nm to 11.0 nm with respect to the interparticle distance between core and satellite Au NPs (Fig. S8). This is probably because that negatively charged DNA scaffold was electrostatically absorbed by positively charged FCS and thus hindered the shrink of DNA scaffold. As a contrast, we prepared PEG-modified core nanoparticles and incubated them in 10% FCS for 6 h, a slightly aggregation in TEM (Fig. S9) and ~12 nm increase of hydrodynamic size of PEG-modified core nanoparticles were observed. Even though PEG is helpful to improve the stabilities of nanoparticles, there were also agglomeration of PEG-modified core nanoparticles after incubation in FCS due to replacement and/or non-specific adsorption, which would also be found from previous report^36^. Therefore, the DNA-directed PEG-CSAuNAs had good stability in serum.

### Cytotoxicity studies of the PEG-CSAuNAs

**Figure 6:**
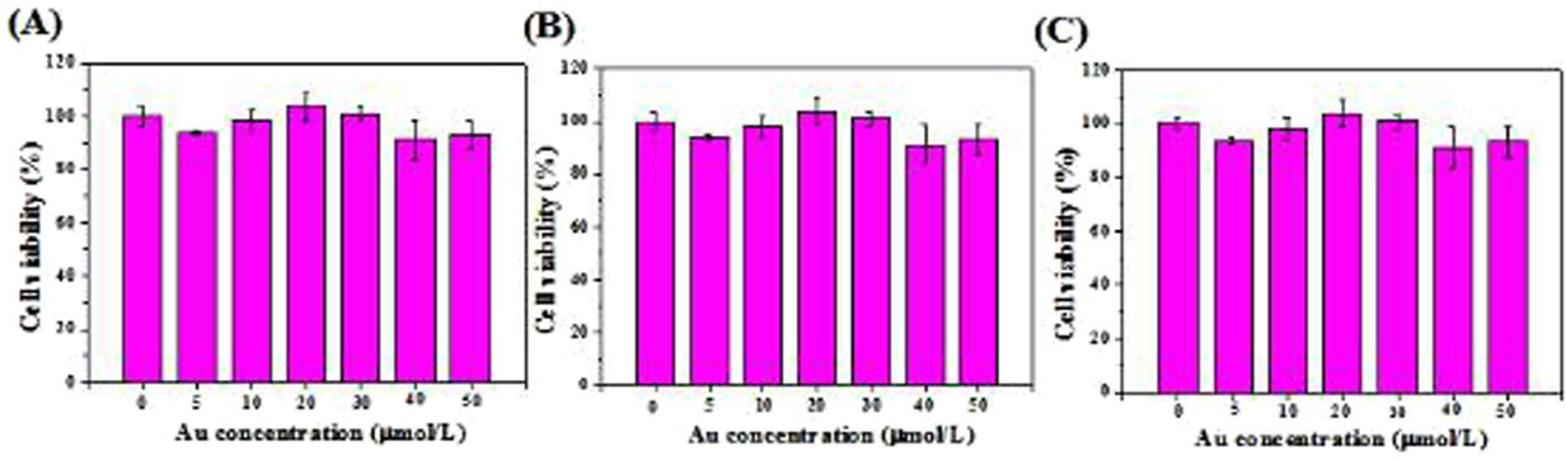
*In vitro* cytotoxicity studies of the PEG-CSAuNAs performed on HK-2 cell lines by CCK-8 assays. Cells were exposed to different concentrations of the PEG-CSAuNAs (5~50 µmol/L Au) for (**A**) 6 h, (**B**) 12 h and (**C**) 24 h, respectively.

Figure 6 shows histogram plots of *in vitro* cytotoxicity of the PEG-CSAuNAs incubated with human renal tubular epithelial (HK-2) cells. As shown in Fig. 6, the viability of HK-2 cells cultured with the PEG-CSAuNAs for 6, 12 and 24 h was more than 90%. Thus, the PEG-CSAuNAs in a wide concentration range from 5 to 50 µmol/L had no obvious cytotoxicity.

### Cell targeting of the NACS-PEG-CSAuNAs and NACS-PEG-AuNPs

**Figure 7:**
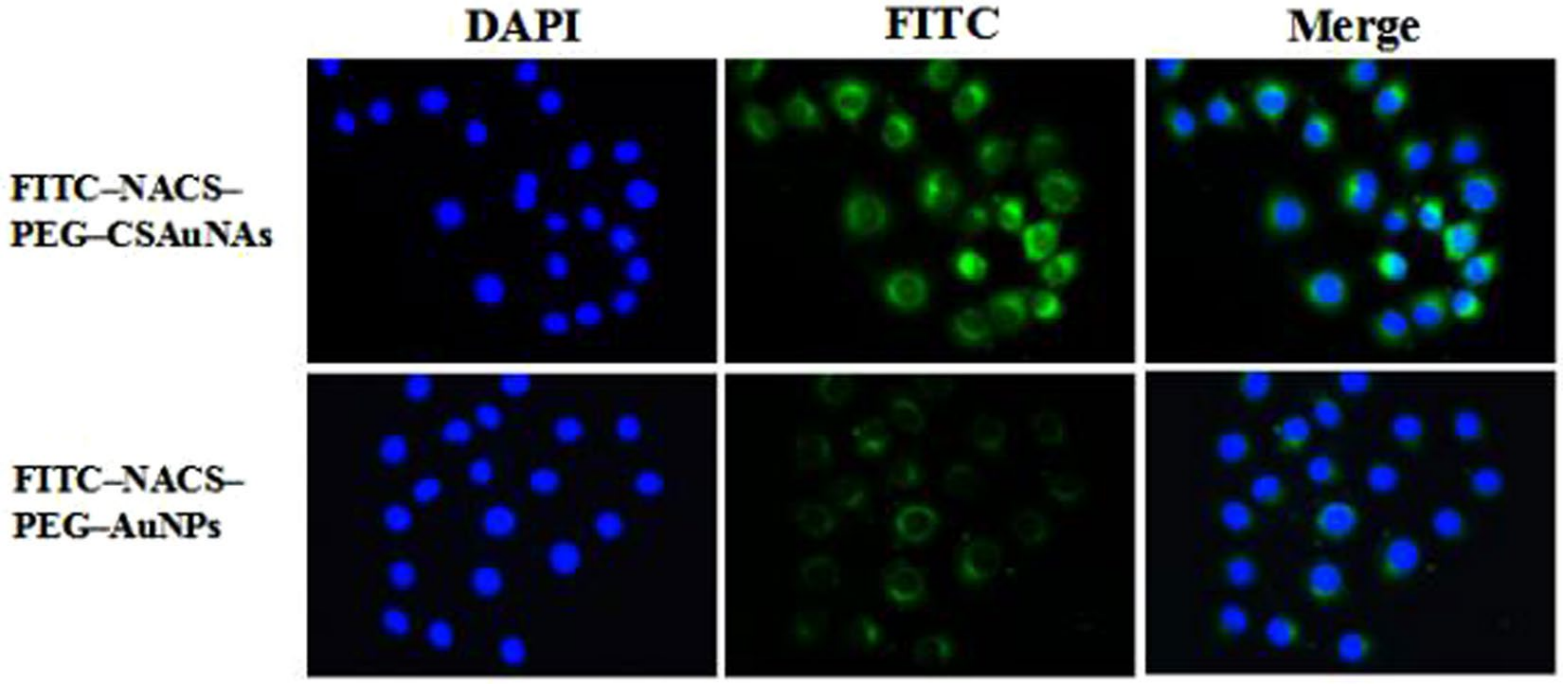
Comparison of cellular uptake of F ITC-NACS-PEG-CSAuNAs and FITC-NACS-PEG-AuNPs.

To visualize cell targeting, the PEG-CSAuNAs or PEG-AuNPs were first conjugated with NACS and FITC. Then, tubular epithelial cells were explored to investigate cell targeting of the PEG-CSAuNAs or PEG-AuNPs. Cell targeting of the PEG-CSAuNAs or PEG-AuNPs towards tubular epithelial cells was performed through interaction between NACS and megalin receptor of tubular epithelial cells, which was partly ascribed to the glucosamine of NACS^40, 41^ and might be an active cellular uptake process. After co-cultured with PEG-CSAuNAs or PEG-AuNPs for 3 h, the results demonstrated that FITC-NACS-PEG-CSAuNAs was more easily taken in than FITC-NACS-PEG-AuNPs (Fig. 7). To further explore the possible mechanism of cell uptake, FITC-NACS-PEG-CSAuNAs were treated with EDTA which is a competitor of calcium-dependent endocytosed pathway^42^. As shown in Fig. S10, apparent intracellular fluorescence could be discerned in the cytoplasm in the absence of EDTA, but it would disappear in the presence of EDTA. This suggested that the NACS-PEG-CSAuNAs also had good cell targeting, which will offer an avenue for *in vivo* targeted therapy toward renal diseases.

### Fluorescence imaging of FITC-NACS-PEG-CSAuNAs and FITC-NACS-PEG-AuNPs *in vivo*

**Figure 8:**
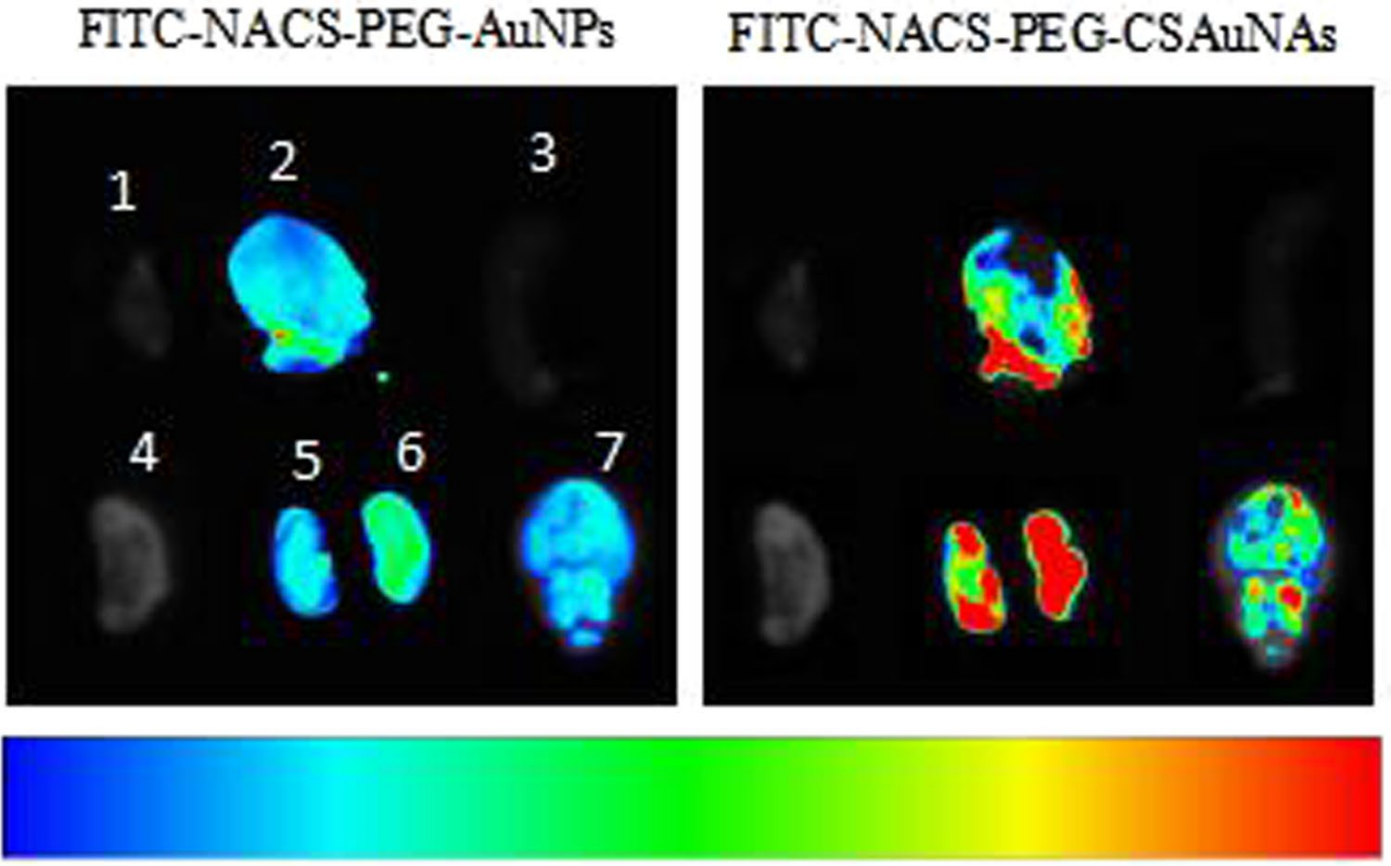
Fluorescence imaging of FITC-NACS-PEG-CSAuNAs and FITC-NACS-PEG-AuNPs *in vivo* (1, heart; 2, liver; 3, spleen; 4, lung; 5, left kidney; 6, right kidney; 7, brain).

To evaluate *in vivo* biodistribution and kidney targeting efficacy of FITC-NACS-PEG-CSAuNAs and FITC-NACS-PEG-AuNPs, male Balb/c mice were intravenously injected FITC-NACS-PEG-CSAuNAs or FITC-NACS-PEG-AuNPs, and sacrificed at 3 h post-injection. Major organs were collected to exposed to imaging system. Figure 8 indicated selective accumulation of FITC-NACS-PEG-CSAuNAs in kidney, whereas no significantly bright fluorescence was observed in all other tissues. Whereas, no special fluorescence distributed in kidneys after administrated with FITC-NACS-PEG-AuNPs. These suggested that FITC-NACS-PEG-CSAuNAs exhibited higher renal retention and apparent difference in different organs than FITC-NACS-PEG-AuNPs, which might be attributed to the good stability of NACS-PEG-CSAuNAs.

## Conclusions

In conclusion, we have constructed novel CSAuNAs with core-satellite structure by a hierarchical DNA-directed self-assembly strategy, in which the CSAuNAs were modified with PEG, FITC and NACS. The satellite Au NPs number in the CSAuNAs could be effectively adjusted through varying the ratios of core-AuNPs-ssDNA and satellite-AuNPs-ssDNAc. The PEG-functionalized CSAuNAs not only possessed highly salt-bearable ability but also could resist the degradation of nuclease in serum. Moreover, cell targeting and imaging indicate that the PEG-CSAuNAs have excellent biotargeting and bioimaging capability. And more importantly, *in vivo* fluorescence imaging analysis revealed that PEG-CSAuNAs modified on CSNA could be specifically targeted to kidneys with satisfactory renal retention capability. The highly stabilized PEG-CSAuNAs open a new avenue for Au NAs applications in biomedicine such as bioimaging and targeted therapy.

## Methods

### Materials

1×TBE buffer: tris(hydroxymethyl)aminomethane (Tris, 89 mM), chloroauric acid (HAuCl_4_), disodium ethylenediaminetetraacetic (EDTA, 2 mM), polyethylene glycol (PEG, Mw = 5,000), bis(p-sulfonateophenyl)phenylphosphine dihydrate dipotassium (BSPP), sodium dihydrogen phosphate (NaH_2_PO_4_.2H_2_O), disodium phosphate dodecahydrate (Na_2_HPO_4_.12H_2_O), fluorescein isothiocyanate (FITC), dithiothretol (DTT) and sodium borohydride (NaBH_4_) were obtained from Sigma-Aldrich (USA). Sodium dodecylsulfate (SDS), sodium citrate (Na_3_C_6_H_5_O_7_), sodium chloride (NaCl) were analytical grade reagent from Guangdong Guanghua Chemical Reagent Co. (China). DNA oligonucleotides were synthesized by Sangon Biotech. Co. (China). Fetal calf serum (FCS), 4′,6-diamidino-2-phenylindole (DAPI), renal tubular epithelial cells and 0.05% trypsin from American Type Culture Collection. N-acetylation chitosan (NACS) was prepared according to the previous method in our group^43^. All the reagents were used as received without further purification. Milli-Q water (>18.0 MΩ cm) was used to prepare all aqueous solutions.

### Self-assembly of CSAuNAs

Firstly, ssDNA were activated using 0.1 M DTT and purified through Nap-5 columns before use. Meanwhile, core Au NPs with the average diameter of approximately 18.7 nm were prepared according to the literature^44^ with certain modification while the ~3.9 nm satellite Au NPs were synthesized through the previously reported NaBH_4_ reduction method^45^. 10 mM phosphate buffer solution (PBS, pH = 7.3) prepared by mixing 72 mL 200 mM Na_2_HPO_4_ and 28 mL 200 mM NaH_2_PO_4_ solution was used for the following self-assembly. Then, core Au NPs were conjugated with ssDNA at the molar ratio of 1:200 in 0.1% SDS, 10 mM PBS and 50 mM NaCl solution. The concentration of NaCl solution was gradually increased up to 200 mM within 10 h to facilitate the effective conjugation between multiple ssDNA and core Au NPs. The mixture were gently shaken for 12 h and centrifuged and redispersed in 10 mM PBS. Meanwhile, satellite Au NPs were coupled with ssDNAc at 1:1 molar ratio in 0.1% SDS, 10 mmol/L PBS and 50 mmol/L NaCl solution and incubated at 4 °C for 10 h. After that, the mixture was dialyzed in 10 mmol/L PBS to obtain the purified satellite-AuNPs-ssDNAc conjugates. Finally, core-AuNPs-ssDNA and satellite-AuNPs-ssDNAc were mixed at the molar ratio of 1:40, 1:80, 1:160 and 1:320. The mixture was mildly stirred under room temperature for 24 h and separated through centrifugation to remove the unbounded satellite-AuNPs. The red precipitate were redispersed and reserved at 4 °C for further use.

### PEG modification and fluorescence labeling of CSAuNAs and AuNPs

In a typical procedure for the PEG modification of CSAuNAs and AuNPs, HS-PEG-COOH was first added to the CSAuNAs(1:160) and AuNPs. Then, the mixture was shook sufficiently and incubated at 4 °C for 9 h. Finally, the PEG-CSAuNAs and PEG-AuNPs were obtained by centrifugation.

In a typical procedure for the fluorescence labeling of CSAuNAs and AuNPs, HS-PEG-COOH and HS-PEG-FITC (1:1 molar ratio) were first added to the CSAuNAs(1:160) and AuNPs. Then, the mixture was shook sufficiently and incubated at 4 °C for 9 h. Finally, the FITC-PEG-CSAuNAs and FITC-PEG-AuNPs was obtained by centrifugation.

### Cytotoxicity assessment of CSAuNAs

To examine the cytotoxicity induced by PEG-CSAuNAs, human renal tubular epithelial cells were cultured in Dulbecco’s Modified Eagle Medium (DMEM) medium supplemented with 10% heat-inactivated FCS at 37 °C in a humidified environment of 5% CO_2_. The culture medium was replaced every 48 to 72 h. Then, cells at approximately 80% confluence were trypsinized using 0.05% trypsin/0.02% EDTA solution. Next, cells were seeded into three 96-well plates at a density of 5,000 cells per well. After 24 h, old medium was discarded and followed by replacement with fresh DMEM serum-free medium. Finally, cells were subjected to PEG-CSAuNAs of a wide concentration range from 5 to 50 μmol/L and incubated for 6, 12 and 24 h, respectively, in which the cells untreated with PEG-CSAuNAs were used as the control groups. 10 μL cell counting kit-8 (CCK-8) was added into each well and cells were further maintained for another 2 h. The optical density (OD) was measured with a microplate reader at the wavelength of 450 nm.

### Cell targeting of FITC-NACS-PEG-CSAuNAs and FITC-NACS-PEG-AuNPs

Firstly, soluble N-acetylation chitosan was conjugated with FITC-PEG-CSAuNAs or FITC-PEG-AuNPs. Then, tubular epithelial cells were cultured in 6-well-plates and divided into three groups contained with FITC-PEG-AuNPs, FITC-PEG-CSAuNAs and FITC-PEG-CSAuNAs contained with 0.2 mg/ml EDTA. Then, all group cells co-cultured in 37 °C for 2 h. Theses cells were washed using 50 mM glycine and 100 mM NaCl. Finally, these cells were dyed by DAPI (blue fluorescence).

### Fluorescence imaging of FITC-NACS-PEG-CSAuNAs and FITC-NACS-PEG-AuNPs *in vivo*

Male Balb/c mice were intravenously administered FITC-NACS-PEG-CSAuNAs or FITC-NACS-PEG-AuNPs and sacrificed 3 h after injection. Major organs were collected and exposed to a imaging system equipped with an excitation at 470 nm and an emission at 535 nm.

### Characterizations

Transmission electron microscopy (TEM) was carried out with a Hitachi H-7500 microscope operated at 80 kV. UV-visible (UV-vis) absorption spectra were performed using a Hewlett-Packard 8452 diode array spectrometer (U-3010). Particle size distribution and zeta potential were recorded by Malvern Nano-ZS (UK). Agarose gel electrophoresis (AGE) samples were acquired by using Powersupplies PowerPac HV (Bio-Rad, American) performed at 80 V for 50 min. Fluorescence spectra were measured by LS 55 Fluorescence Spectrometer (PerkinElmer, American). *In vivo* fluorescence imaging was scanned with Bruker FX PRO imaging system (Rochester, NY, USA).

## Electronic supplementary material

Supplementary information

## References

[1] Daniel MC, Astruc D (2004). Gold nanoparticles: assembly, supramolecular chemistry, quantumsize-related properties, and applications toward biology, catalysis, and nanotechnology. Chem. Rev..

[2] Liu AB, Ye B (2013). Application of gold nanoparticles in biomedical researches and diagnosis. Clin. Lab..

[3] Zhou C, Hao GY, Zheng J (2012). Near-infrared emitting radioactive gold nanoparticles with molecular pharmacokinetics. Angew. Chem. Int. Ed..

[4] Brown SD, Nativo P, Wheate NJ (2010). Gold nanoparticles for the improved anticancer drug delivery of the active component of oxaliplation. J. Am. Chem. Soc..

[5] Perrault SD, Walkey C, Chan WCW (2009). Mediating tumor targeting efficiency of nanoparticles through design. Nano Lett..

[6] Lai XD, Tan LS, Hu JQ (2017). Coordinatively self-assembled luminescent gold nanoparticles: fluorescence turn-on system for high-efficiency passive tumor imaging. ACS Appl. Mater. Inter..

[7] Hainfeld JF, Dilmanian FA, Smilowitz HM (2010). Gold nanoparticles enhance the radiation therapy of a murine squamous cell carcinoma. Phys. Med. Biol..

[8] Wang LV, Hu S (2012). Photoaacoustic tomography: *in vivo* imaging from organelles to organs. Science.

[9] Bhamidipati M, Febris L (2017). Multiparametric assessment of gold nanoparticle cytotoxicity in cancerous and healthy cells: the role of size, shape, and surface chemistry. Bioconjugate Chem..

[10] Yang X, Yang MX, Xia YN (2015). Gold nanomaterials at work in biomedicine. Chem. Rev..

[11] Khlebtsov N, Dykmana L (2011). Biodistribution and toxicity of engineered gold nanoparticles: a review of *in vitro* and *in vivo* studies. Chem. Soc. Rev..

[12] Potenza MAC, Krpetić Ž, Dawson KA (2017). Detecting the shape of anisotropic gold nanoparticles in dispersion with single particle extinction and scattering. Nanoscale.

[13] Niidome T, Yamagata M, Niidome Y (2006). PEGmodified gold nanorods with a stealth character for *in vivo* applications. J. Control. Release.

[14] Yan Y, Shan HY, Hu JQ (2015). Internal-modified dithiol DNA-directed Au nanoassemblies: geometrically controlled self-assembly and quantitative surface-enhanced Raman scattering properties. Sci. Rep..

[15] Fan JA, He Y, Liu DR (2011). DNA-enabled self-assembly of plasmonic nanoclusters. Nano Lett..

[16] Retnakumari A, Setua S, Koyakutty M (2010). Molecular-receptor-specific, non-toxic, near-Infrared-emitting Au cluster-protein nanoconjugates for targeted cancer imaging. Nanotechnology.

[17] Sharma H, Dormidontova EE (2017). Lipid nanodisc-templated self-assembly of gold nanoparticles into strings and rings. ACS Nano..

[18] Kim JW, Kim JH, Deaton R (2011). DNA-linked nanoparticle building blocks for programmable matter. Angew. Chem. Int. Ed..

[19] Seeman NC (2003). DNA in a material world. Nature.

[20] Aldaye FA, Sleiman HF (2007). Dynamic DNA templates for discrete gold nanoparticle assemblies: control of geometry, modularity, write/erase and structural switching. J. Am. Chem. Soc..

[21] Sharma J, Chhabra R, Yan H (2006). DNA-templated self-assembly of two dimensional and periodical gold nanoparticle arrays. Angew. Chem. Int. Ed..

[22] Barrow SJ, Funston AM, Mulvaney P (2013). DNA-directed self-assembly and optical properties of discrete 1D, 2D and 3D plasmonic structures. Nano Today.

[23] Coomber D, Bartczak D, Stulz E (2010). Programmed assembly of peptide-functionalized gold nanoparticles on DNA templates. Langmuir.

[24] Lan X, Chen Z, Wang QB (2013). DNA-directed gold nanodimers with tailored ensemble surface-enhanced Raman scattering properties. ACS Appl. Mater. Inter..

[25] Cheng YF, Lai XD, Hu JQ (2013). Al^3+^-directed electrostatic self-assembly and their surface plasmon resonance properties of Au nanocrystals. RSC Adv..

[26] Ma XY, Huh J, Sim SJ (2016). Gold nanocrystals with DNA-directed morphologies. Nat. Commun..

[27] He Y, Peng RF (2014). Luminol functionalized gold nanoparticles as colorimetric and chemiluminescent probes for visual, label free, highly sensitive and selective detection of minocycline. Nanotechnology.

[28] Valentini P, Fiammengo R, Pompa PP (2013). Gold-nanoparticle-based colorimetric discrimination of cancer-related point mutations with picomolar sensitivity. ACS Nano..

[29] Tripathy SK, Woo JY, Han CS (2012). Surface-plasmon-based colorimetric detection of Cu(II) ions using label-free gold nanoparticles in aqueous thiosulfate systems. Nanotechnology.

[30] Seferos DS, Prigodich AE, Mirkin CA (2009). Polyvalent DNA nanoparticle conjugates stabilize nucleic acids. Nano Lett..

[31] Gibson MI, Danial M, Klok HA (2011). Sequentially Modified, Polymer-Stabilized Gold Nanoparticle Libraries: Convergent Synthesis and Aggregation Behavior. ACS Comb. Sci..

[32] Liu JB, Yu MX, Zheng J (2013). PEGylation and Zwitterionization: Pros and Cons in the renal clearance and tumor targeting of near-IR-emitting gold nanoparticles. Angew. Chem. Int. Ed..

[33] Knop K, Hoogenboom R, Schubert US (2010). Poly(ethylene glycol) in drug delivery: pros and cons as well as potential alternatives. Angew. Chem. Int. Ed..

[34] Hutter E, Boridy S, Maysinger D (2010). Microglial response to gold nanoparticles. ACS Nano..

[35] Gref R, Domb A, Langer R (2012). The controlled intravenous delivery of drugs using PEG-coated sterically stabilized nanospheres. Adv. Drug Deliver. Rev..

[36] Kamimura M, Kanayama N, Nagasaki Y (2011). Near-infrared (1550 nm) *in vivo* bioimaging based on rare-earth doped ceramic nanophosphors modified with PEG-b-poly(4-vinylbenzylphosphonate). Nanoscale.

[37] Zhang J, Malicka J, Lakowicz JR (2004). Oligonucleotide-displaced organic monolayer-protected silver nanoparticles and enhanced luminescence of their salted aggregates. Anal. Biochem..

[38] Pamies R, Cifre JGH (2014). Aggregation behaviour of gold nanoparticles in saline aqueous media. J. Nanopart. Res..

[39] Gleave ME, Monia BP (2005). Antisense therapy for cancer. Nat. Rev. Cancer.

[40] Yuan ZX, Zhang ZR, Luan CT (2009). Specific renal uptake of randomly 50% N-acetylated low molecular weight chitosan. Mol. Pharmaceutics.

[41] Orlando RA, Rader K, Farquhar MG (1998). Megalin is an endocytic receptor for insulin. J. Am. Soc. Nephrol..

[42] Qiao HZ, Sun MJ, Ping QN (2014). Kidney-specific drug delivery system for renal fibrosis based on coordination-driven assembly of catechol-derived chitosan. Biomaterials.

[43] Li M, Tan LS, Hu JQ (2016). Hydrosoluble 50% N-acetylation-thiolated chitosan complex with cobalt as a pH-responsive renal fibrosis targeting drugs. J. Biomater. Sci. Polym. Ed..

[44] Geng JL, Li K, Liu B (2012). Conjugated polymer and gold nanoparticle Co-loaded PLGA nanocomposites with eccentric internal nanostructure for dual-modal targeted cellular imaging. Small.

[45] Krpetić Z, Singh I, Graham D (2012). Directed assembly of DNA-functionalized gold nanoparticles using Pyrrole-Imidazole Polyamides. J. Am. Chem. Soc..

