# Supplementary material for: Highly Stabilized Core-Satellite Gold Nanoassemblies in Vivo: DNA-Directed Self-Assembly, PEG Modification and Cell Imaging
Source: Sci Rep. 2017 Aug 17;7:8553. doi: 10.1038/s41598-017-08903-0 (PMC5561241; doi:10.1038/s41598-017-08903-0)
Supplement: Supplementary file 1 — Supplementary information [file 41598_2017_8903_MOESM1_ESM.doc]

**Supporting Information**

**Highly Stabilized Core–Satellite Gold Nanoassemblies in Vivo: DNA–Directed Self–Assembly, PEG Modification and Cell Imaging**

*Liangfeng Tang^1,3,‡^, Guiping Yu^1,3,‡^, Lishan Tan^2‡^, Min Li^1^, Xiulong Deng^1^, Jianyu Liu,^1^,* Aiqing Li,^2^,* Xuandi Lai^1^ & Jianqiang Hu^1,3^,**

^1^ Department of Chemistry, College of Chemistry and Chemical Engineering, South China University of Technology, Guangzhou, 51040, China. E–mail: [jqhusc@scut.edu.cn](mailto:jqhusc@scut.edu.cn); [jyliu@scut.edu.cn](mailto:jyliu@scut.edu.cn).

^2^ State Key Laboratory of Organ Failure Research, National Clinical Research Center for Kidney Disease, Nanfang Hospital, Southern Medical University, Guangzhou 510515, China. E–mail: liaiqing@smu.edu.cn.

^3^ State Key Laboratory of Pulp and Paper Engineering, South China University of Technology, Guangzhou, 510640, China.

^‡^ Liangfeng Tang, Guiping Yu and Lishan Tan equally contributed to this work.

**Table S1 |** Oligonucleotide sequences used in this study.

| **Name** | **Sequence** |
| --- | --- |
| ssDNA | **5**´**–**HS–(CH_2_)_6_ ATC CTG ACA TCG GCA CGA GTA TTT CTA CCA TGT ATC**–3'** |
| ssDNAc | **5**´**–**HS–(CH_2_)_6_ GAT ACA TGG TAG AAA TAC TCG TGC CGA TGT CAG GAT**–3'** |

**Figure S1**


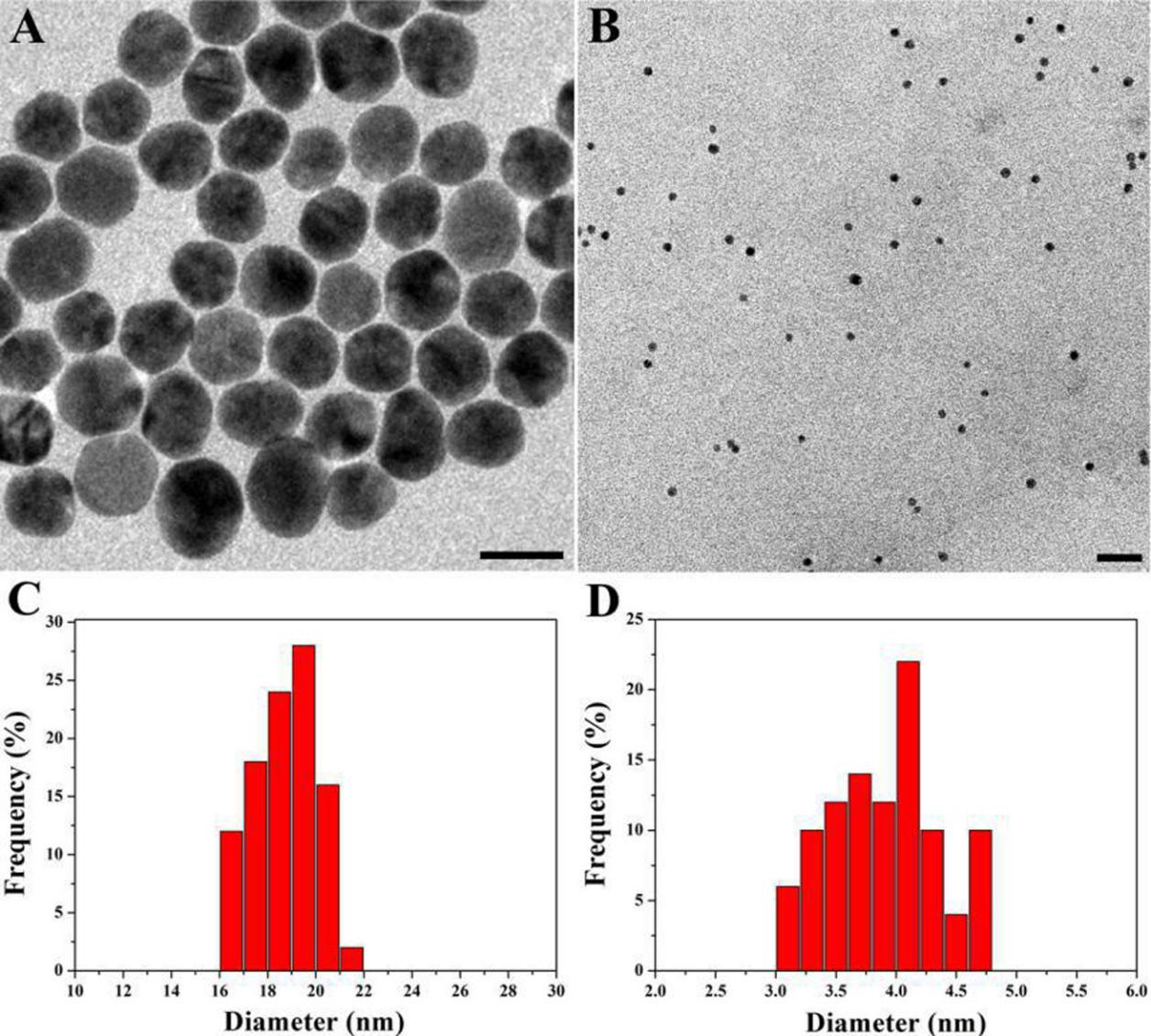


**Figure S1** **|** TEM images and size distribution histograms of (A,C) core Au NPs and (B,D) satellite Au NPs. The size distribution histograms were depicted through evaluating the sizes of 100 NPs in the TEM images. Scale bar: 20 nm.


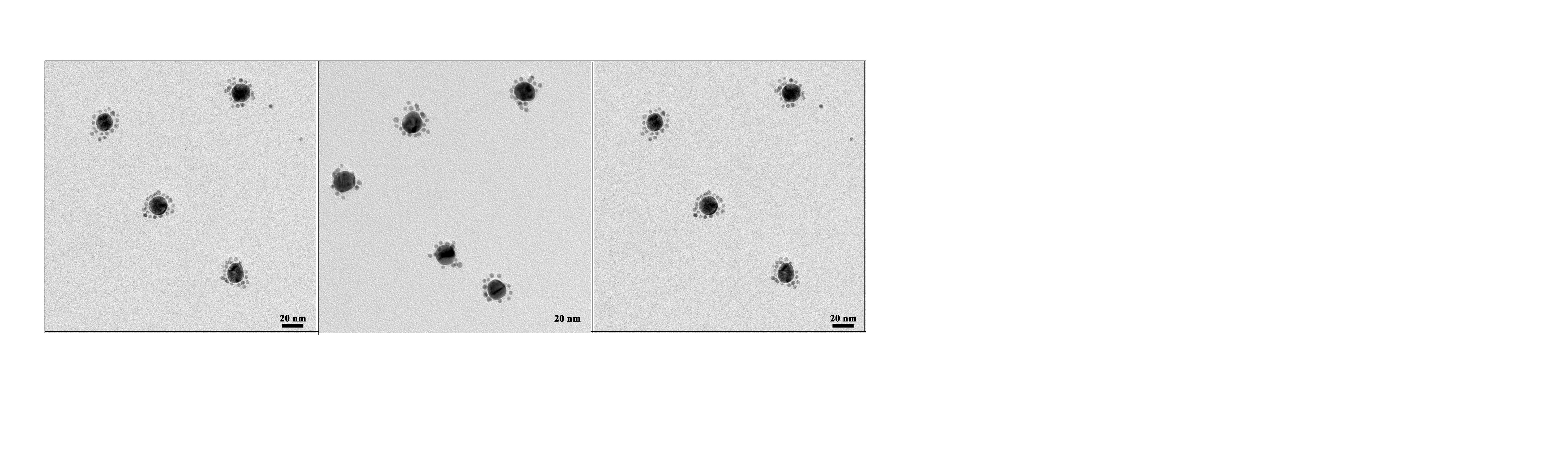
（）（（））

**Figure S2**


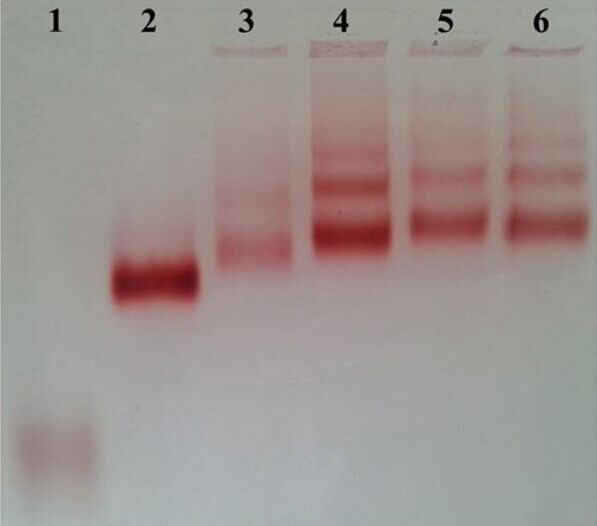


**Figure S2** | Gel electropherogram of the Au NPs amd CSAuNAs prepared using different molar ratios of core–AuNPs–ssDNA to satellite–AuNPs–ssDNAc. Lanes 1–6 corresponded to satellite Au NPs, core Au NPs and CSAuNAs assembled with the molar ratios of 1:40, 1:80, 1:160 and 1:320, respectively.

**Figure S3**


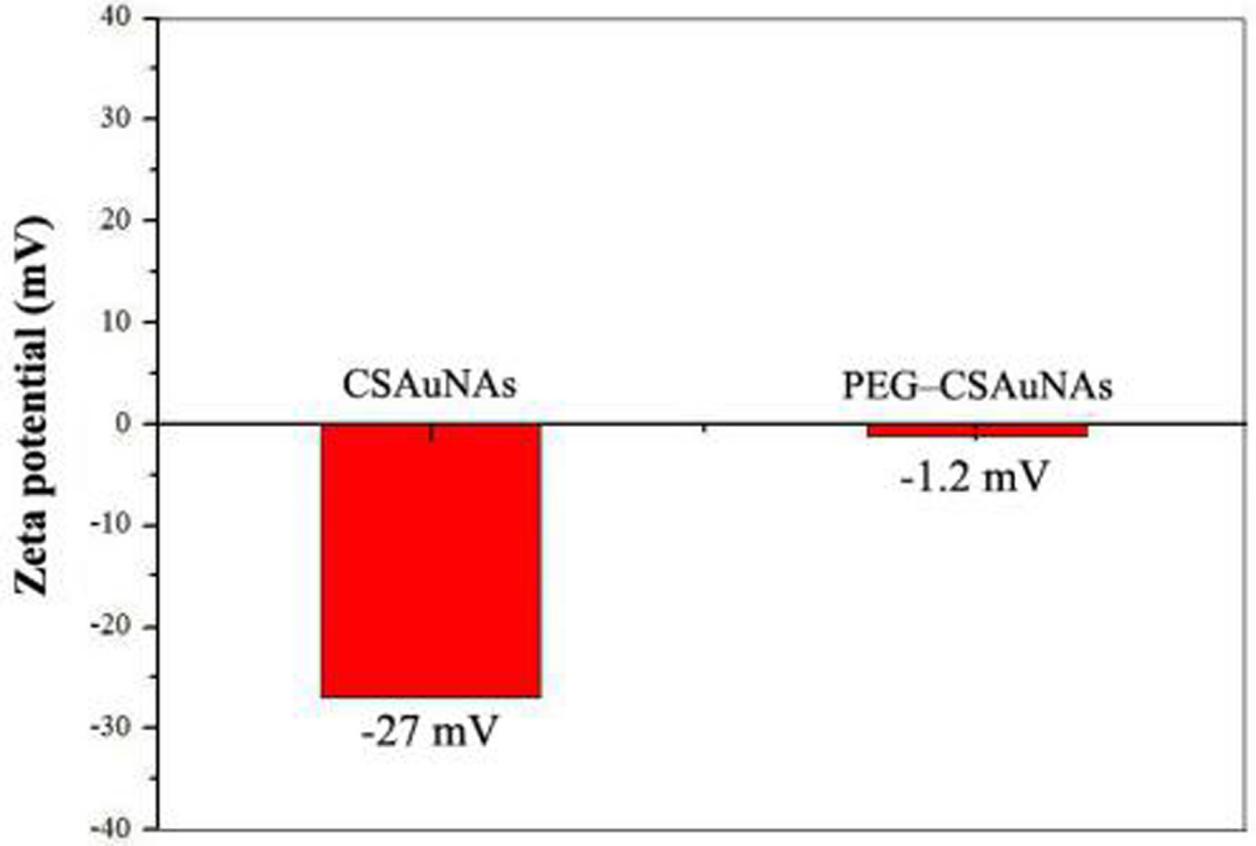


**Figure S3** | Zeta–potential plot of the CSAuNAs and PEG–CSAuNAs.

**Figure S4**


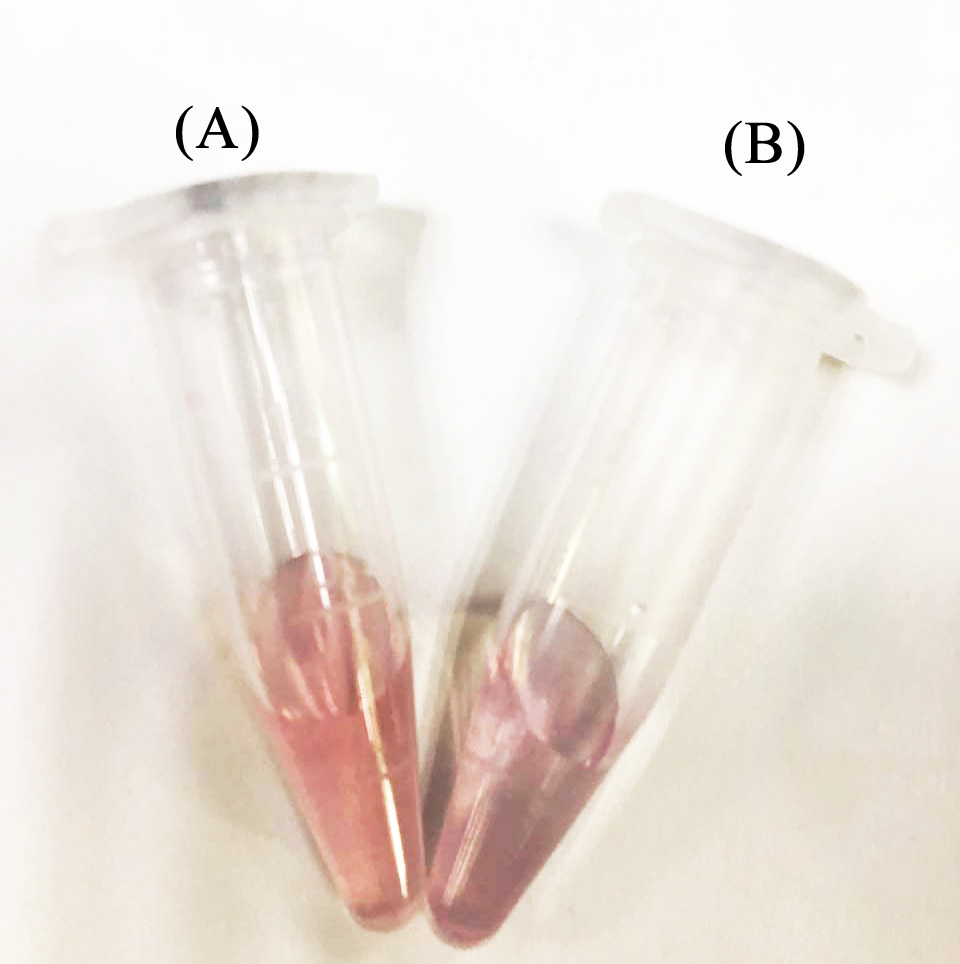


**Figure S4 |** Digital photos of the (**A**) PEG–CSAuNAs and (**B**) PEG–core AuNPs after incubation with normal saline for 30 min.

**Figure S5**


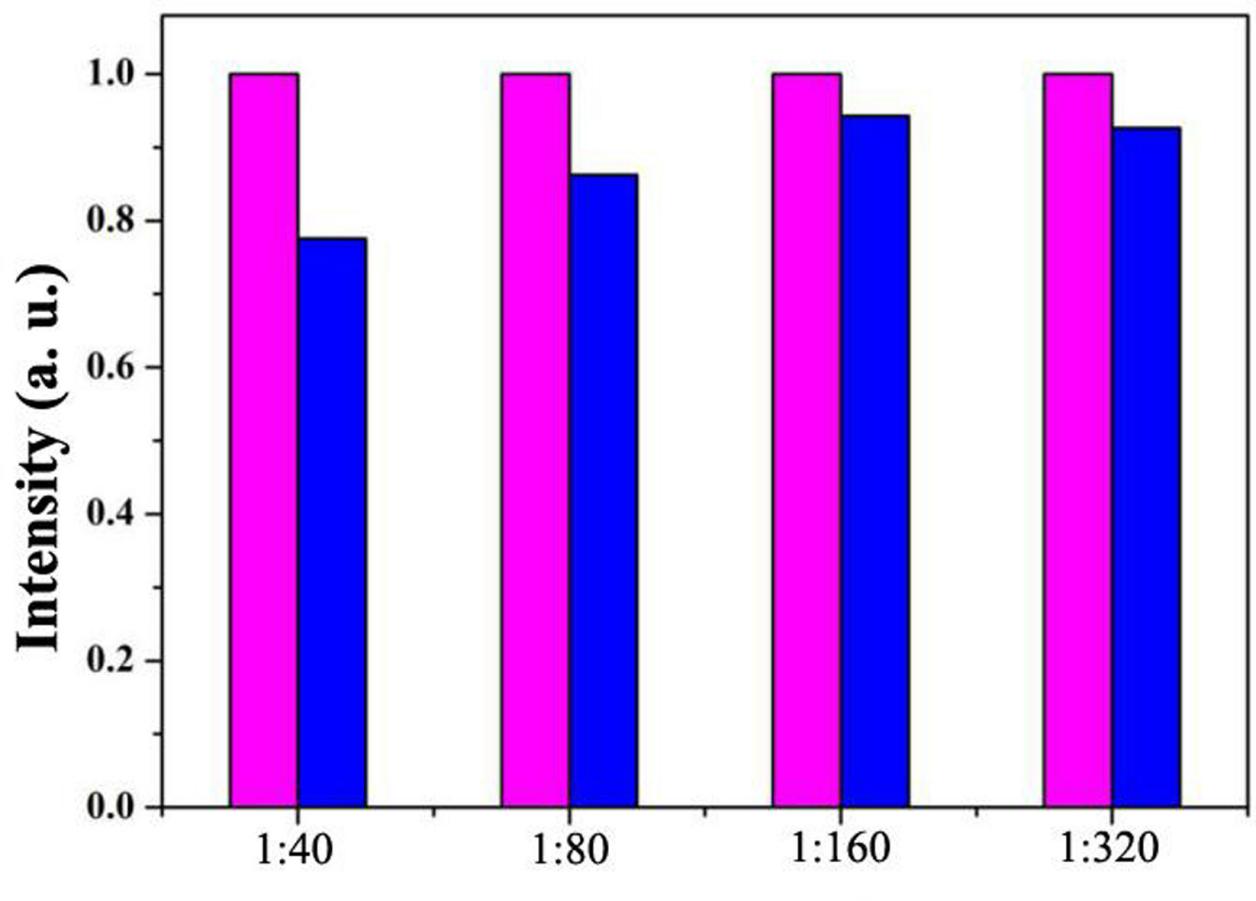


**Figure S5 |** Histogram plots of the maximum absorption intensities of the CSAuNAs prepared at the molar ratios between core–AuNPs–ssDNA and satellite–AuNPs–ssDNAc of 1:40, 1:80, 1:160 and 1:320 before (pink bars) and after (blue bars) incubation with normal saline for 5 days. The maximum absorption intensities were normalized by setting the maximum absorption intensities of the CSAuNAs as 1.

**Figure S6**


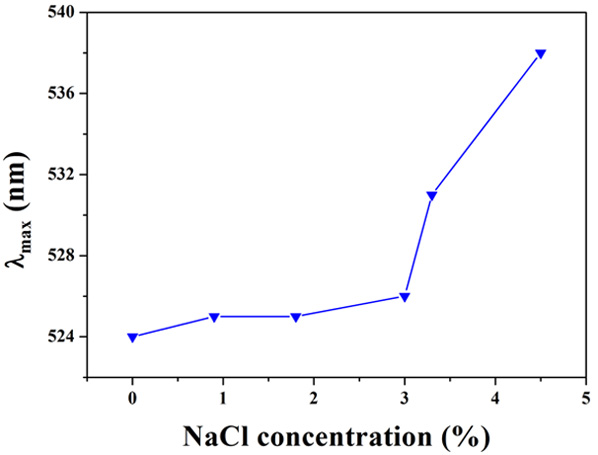


**Figure S6 |** The maximum absorption wavelength plot of the CSAuNAs prepared at the 1:160 molar ratio between core–AuNPs–ssDNA and satellite–AuNPs–ssDNAc after incubation with different NaCl concentrations for 2 h.

**Figure S7**


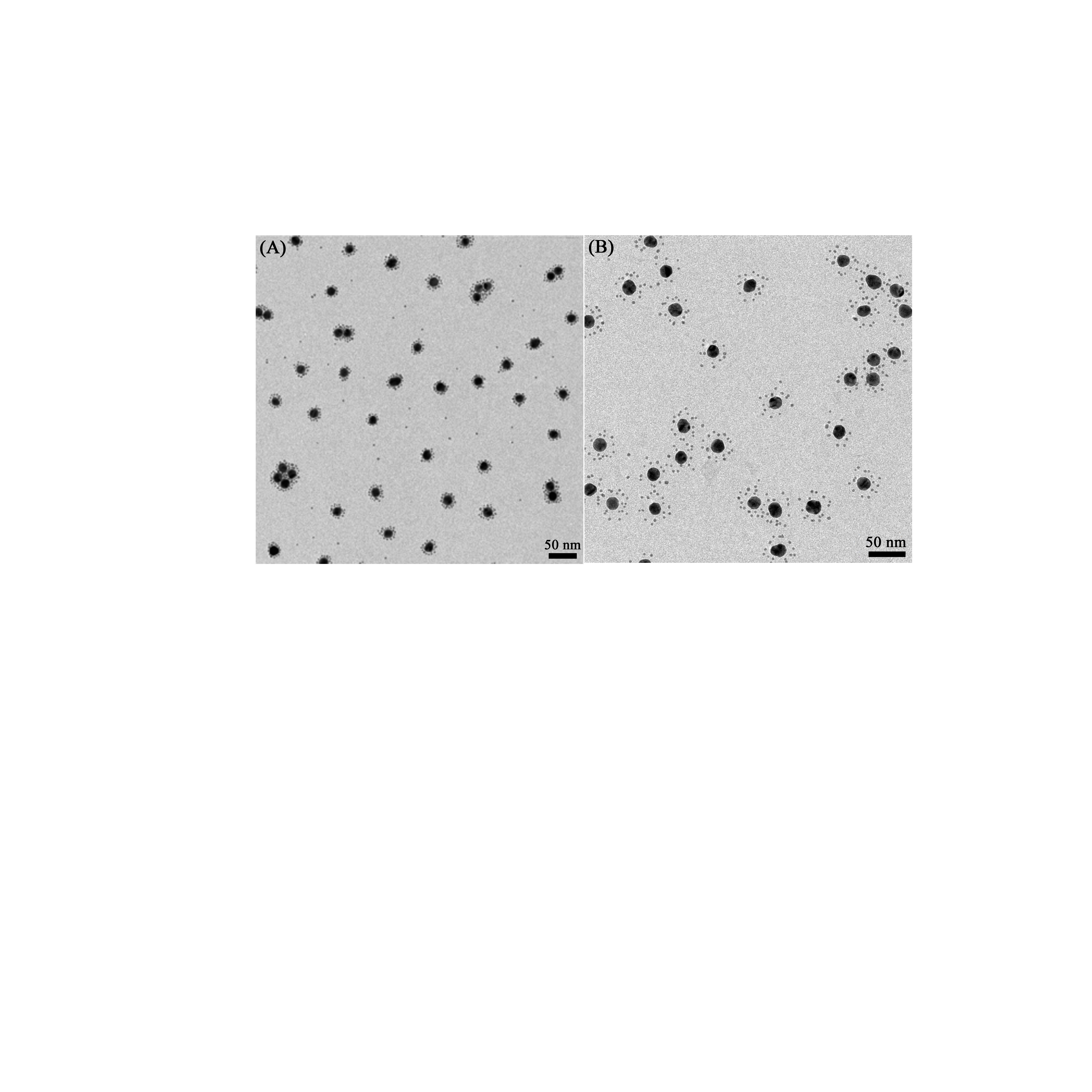


**Figure S7** | TEM images of the PEG–CSAuNAs prepared at the 1:160 molar ratio (A) before and (B) after incubation with 10% FCS for 6 h.

**Figure S8**


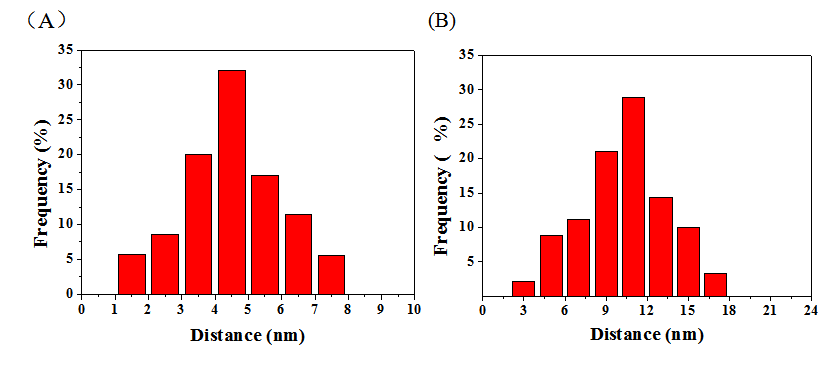


**Figure S8 |** Size distribution histograms of distances between the core and satellite AuNPs (A) before and (B) after incubation with 10% FCS for 6 h. The size distribution histograms were depicted through evaluating the sizes of 100 NPs in the TEM images.

**Figure S9**


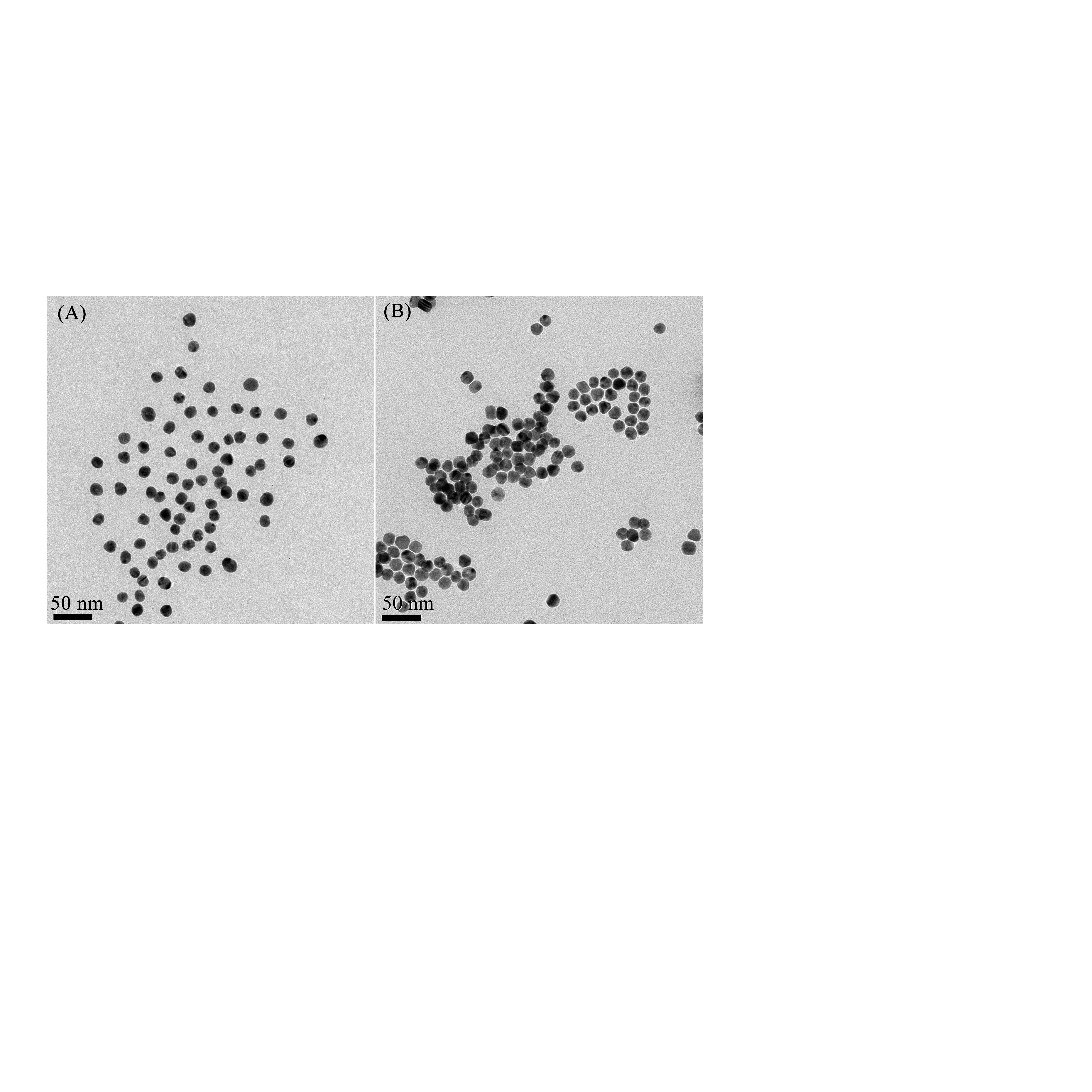


**Figure S9** | TEM images of PEG–modified core nanoparticles (A) before and (B) after incubation in 10% FCS for 6 h.

**Fig S10**


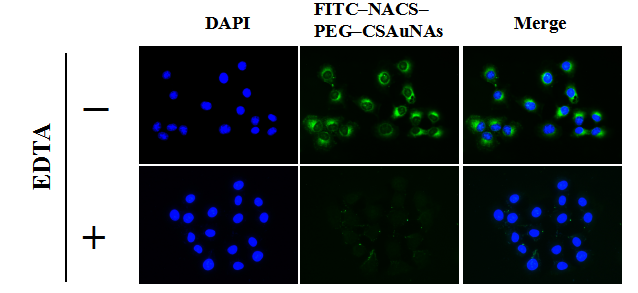


**Figure S10 |** Comparison of cellular uptake of FITC–NACS–PEG–CSAuNAs in the absence or presence of EDTA.
